# An alternative approach to dimension reduction for pareto distributed data: a case study

**DOI:** 10.1186/s40537-021-00428-8

**Published:** 2021-02-25

**Authors:** Marco Roccetti, Giovanni Delnevo, Luca Casini, Silvia Mirri

**Affiliations:** Department of Computer Science and Engineering, University of Bologna, Via Mura Anteo Zamboni 7, 40127 Bologna, Italy

**Keywords:** Deep learning models, Categorical data, Learning space dimensions, *Pareto* analysis, Imbalanced datasets, Dataset coherence analysis, Principal component analysis, Binning, Machine learning

## Abstract

*Deep learning* models are tools for data analysis suitable for approximating (non-linear) relationships among variables for the best prediction of an outcome. While these models can be used to answer many important questions, their utility is still harshly criticized, being extremely challenging to identify which data *descriptors* are the most adequate to represent a given specific phenomenon of interest. With a recent experience in the development of a deep learning model designed to detect failures in mechanical water meter devices, we have learnt that a sensible deterioration of the prediction accuracy can occur if one tries to train a deep learning model by adding specific device descriptors, based on *categorical* data. This can happen because of an excessive increase in the dimensions of the data, with a correspondent loss of statistical significance. After several unsuccessful experiments conducted with alternative methodologies that either permit to reduce the data space dimensionality or employ more traditional machine learning algorithms, we changed the training strategy, reconsidering that categorical data, in the light of a *Pareto analysis*. In essence, we used those categorical descriptors, not as an input on which to train our deep learning model, but as a tool to give a new shape to the dataset, based on the *Pareto* rule. With this data adjustment, we trained a more performative deep learning model able to detect defective water meter devices with a prediction accuracy in the range 87–90%, even in the presence of categorical descriptors.

## Introduction

When data scientists write a book on how a supervised deep learning project is to be structured, what it is lived at the beginning, i.e., when input data are analyzed, takes up only the first third, or so. The bulk of the story, in fact, is usually considered what happens next: with the model *training and validation process*, and then with the *testing* phases of the outcomes [1].

Unfortunately, the first phase of *data analysis* and preparation is almost never considered as a silver bullet, and it often remains an underinvested branch in the deep learning practice. In some sense, our scientific community has not been as effective at developing strategies to construct reliable datasets as we have with those to learn from them. But that needs to change, under the penalty of severe consequences, ranging from inaccurate predictions to the lack of explainability of our models [2, 3]. Further, the progress we need to achieve cannot be in the abundance of data we collect, alone. It should be also in our ability to make sure that our project actually benefits from the particular data we choose. Focusing all into a huge amount of data can be a good premise, in fact; yet, not asking any questions about their usage, role and the value that can be drawn from it, will turn that premise into the first motivation behind a failure. In other words, data is just an initial representation of a situation, but key remains the way we analyze it. We go so far as to say that data is there, mostly to stimulate an accurate analysis. Whether a single piece of data is either descriptive or predictive, or even prescriptive, in nature, is just what can be understood through an in-depth analysis of that data [4–7].

In this context, the target of our study is to demonstrate that putting a proper *data analysis* at the core of a deep learning project can assist one in identifying the most accurate data *descriptors* for that project. While it is well known in fact that, in computing, a *data descriptor* is just a structure containing information that describes data, yet data descriptors for deep learning can span several diverse aspects, as the data’s provenance and type, up to its storage schema. In essence, descriptors encapsulate a basic knowledge about the data, and can thus be used as starting points for the construction of a trustworthy dataset on which a deep learning model can be safely trained.

Along this line, in this paper we describe a deep learning design experience, where we had initially a trouble on developing an appropriate deep learning model able to detect failures in mechanical water meter devices, because we tried to train that model by merging together the *numerical* information relative to water consumption with some device descriptors based on *categorical* information, thus resulting into an explosion in data dimensionality, that soon determined a deterioration of the prediction accuracy [8, 9]. After several unsuccessful experiments conducted with alternative methodologies that either permitted to reduce the data space dimensionality or employed more traditional machine learning algorithms, we changed the training strategy. In essence, we moved towards an accurate *statistical analysis* of the initial data, culminating with the application of an approximation of the 80/20 *Pareto* rule, that made us understand that categorical descriptors could not be part of the contents our model had to learn. Starting from this changed perspective, we devised a new strategy where categorical descriptors were used just as a driver for data selection, rather than being fed as input to the model. This way, we kept under control the dimensionality of the learning space and, at the same time, we achieved satisfying results of model prediction accuracy, in terms of detection of defective devices, reaching values in the range from 87 to 90%.

**Fig. 1:**
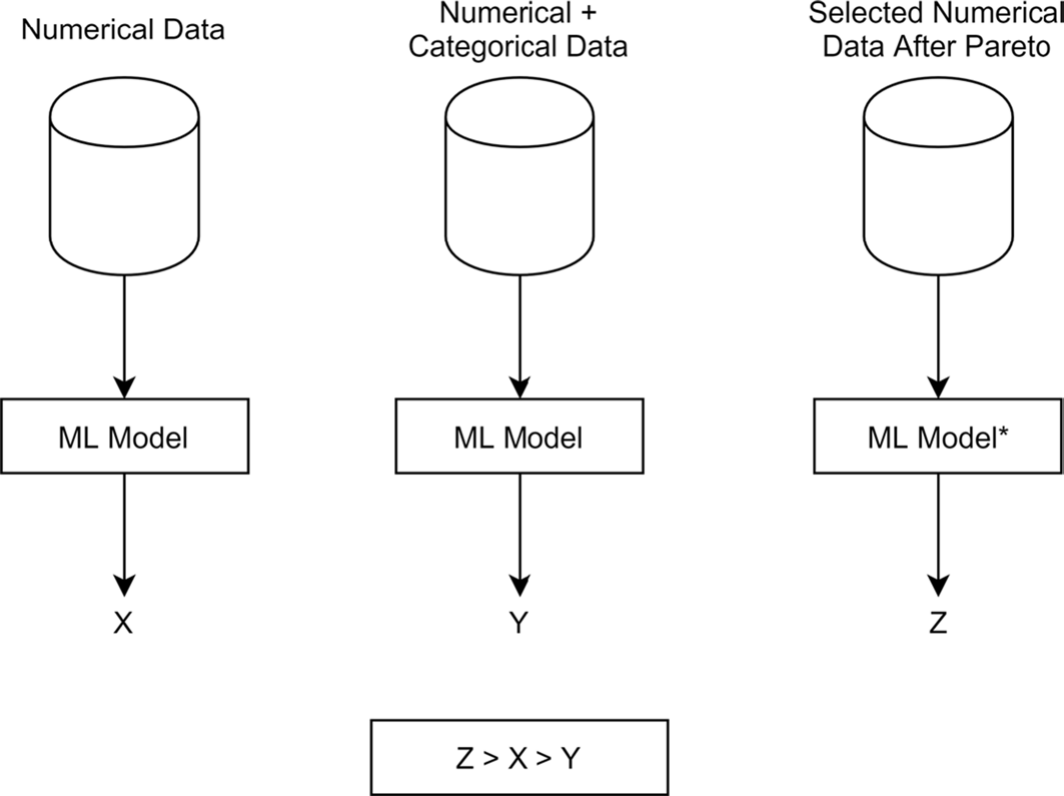
Anticipating the results: prediction accuracies on water meter devices (X = 86%, Y = 83%, Z = 90%)

Anticipating a part of the final results shown in this paper, we introduce Fig. 1, where the *Z*, *X* and *Y* variables represent, respectively, the prediction accuracies, obtained in the following three different situations: (1) The deep learning model is trained only with numerical data (*X*); (2) The deep learning model is trained with a mix of numerical and categorical data (*Y*); (3) The deep learning model is trained with a selection of numerical data, based on a *Pareto* analysis conducted on the categorical data (*Z*).

As portrayed in Fig. 1, and discussed at length in the remainder of the paper, *Z* (90%) outperforms *Y* (83%).

In conclusion, in this paper we demonstrate that it is possible to train a deep learning model that achieves excellent prediction accuracy levels, even in the presence of both numerical and categorical descriptors. This approach was devised to select the training data, based on a *Pareto* analysis conducted on the categorical descriptors, thus avoiding the explosion of the data space dimensions while keeping intact the statistical coherence of the portion of the dataset selected for training. We have provided empirical evidence that this approach maintains its validity even if compared with more traditional space dimension reduction methodologies and classical machine learning algorithms, as well.

The remainder of this paper is structured as follows. In the Section devoted to the *Related work*, the focus is put on what can happen to the dimensions of a learning space when categorical variables are employed, along with a survey on the techniques usually adopted to manage this situation. In the *Methodology* Section: (i) we present the initial dataset on which we have worked, (ii) we illustrate some (unsuccessful) deep learning model training experiments that employed classical techniques to reduce the data dimensionality space, (iii) we describe the approach through which this initial dataset was reshaped, along with an analysis that demonstrates that these data re-adjustment operations do not change the statistical coherence of the selected data, and (iv) we finally illustrate how those data can be used to train a deep learning model. In the Section devoted to describing the *Results*, instead, we present and discuss the results we achieved with our approach. The Section devoted to the *Discussion* supplies: (i) some reflections on the advantages and limitations of our approach, along with a comparison with some alternative machine learning methods, and) (ii) a practical guide on how to use our classifier culminating with the adoption of the additional technique of Bagging, able to further increase the model performances. The final Section provides the *Conclusions* and terminates the paper with some concluding remarks.

## Related work

At the core of this paper lies the question if all types of descriptors should be presented to a deep learning model in a way that is opaque to most of the designers, rather than subjecting them to an accurate data *analysis* before using them for training. In particular, this problem exacerbates when we have to deal with two very different types of data: i.e., *numerical* vs. *categorical*. In fact, while numerical data are measurable in nature, and easily manageable, categorical data, instead, represents a collection of information, that can be divided into groups; e.g., *black* and *white*; and, as such, they can take on numerical values (for example: *1* indicating black, and *2* indicating white), but those numbers do not have a precise mathematical meaning. This is the reason why working with categorical descriptors can easily lead to an increase of the dimensions of the space under investigation. This phenomenon may go so fast that the available data become sparse, with a consequent loss of statistical significance [10].

To understand this phenomenon, take, for example, one of the most common techniques applied to encode *categories* into *numerical* values: the *one-hot encoding* technique [11, 12]. Consider a categorical variable with the following values: *Yes*, *No*, and *Prefer not to say*. They can be encoded with the following vectors {[1, 0, 0], [0, 1, 0], [0, 0, 1]}. This produces a new, three-dimensional space, with a total amount of twenty-seven points. However, the only interesting points remain three, and are orthogonal, equidistant, and sparse. Simply said, we have yielded a three-dimensional vector space, with a new dimension for each original value (*yes*, *no*, *prefer not to say*). Unfortunately, things can even get worse. If we had three categories, each with three values, we would get a nine-dimension space, as this would come with the product of the number of categories *times* the number of possible values [13, 14].

Hence, a general problem can be posed: *how to manage categorical variables, while keeping the dimensionality of the resulting space under control*. To this aim, many statistical techniques have been proposed in literature that are used to face this problem. Typically, the recurrent idea behind all those methods is as follows:

i. Consider a high dimensional categorical space.
ii. Apply a procedure for reducing the number of variables, without loss of information.
iii. Identify new variables with greater meaning, and finally,
iv. Keep as the ultimate target that of maintaining *visible* a lot of points, in this reduced space, to be used as representative examples on which a supervised learning model can be trained.

What is also very common is the fact that the procedure for reducing the dimensionality rests upon the idea of representing the categorical space with a few orthogonal (uncorrelated) variables that capture most of its [15]. In the remainder of this Section, we are going to provide a few details on the principal techniques of this family. Before beginning with this review, we briefly anticipate here that our method will be different. We will avoid to use *categorical* descriptors as input to the model to be trained. Instead, they will be used as a driver for data selection, thus eliminating, from the start, the need for a dimensionality reduction of the categorical space.

Among the traditional methods mentioned above, probably, the *Correspondence Analysis* (with all its variants) is the most known one. Akin to the *Principal Component Analysis*, the Correspondence Analysis (or CA) provides a solution for projecting a set of data onto lower-dimensional plots. Essentially, CA aims at visualizing the rows and the columns of a *contingency table* as points in a low-dimensional space, so that a global view of the data is made available, yet easily interpretable [16–18]. Identically derived from the *Principal Component Analysis*, we have the *CATegorical Principal Components Analysis* (or CATPCA). Here, again, the final goal is to reduce the data dimensions by projecting them onto a low-dimensional plane, with the *plus* that the relationships among observed variables are not assumed to be linear [19].

Of interest in this field, it is also the so-called *Multi-Dimensional Scaling* (MDS) technique. Technically speaking, MDS is used to translate information about the *pairwise distances* among a set of *n* objects into a configuration of *n* points, mapped into an abstract Cartesian space. In essence, this technique is proven to be useful to display the information contained in a distance matrix, while providing a form of non-linear dimensionality reduction [20, 21]. Sometimes, also some kind of *structural equation modeling* is employed to individuate groups, or subtypes, in the case of multivariate categorical data. These are called *latent classes*, as detailed in the following references [22–25].

Another interesting technique, in the context of multivariate statistics, is that of *Binning*. Here the target is somewhat different, since, at the basis, we have a form of data quantization. Essentially, all the data values falling into a given interval (the *bin*, indeed) are all replaced by a single representative value. A typical example, which is provided to explain this technique, is that of representing the ages of a group of people with intervals of consecutive years, rather than with each single age value [26]. Needless to say, going for *binning* is a delicate choice, since some pieces of information can come sacrificed. Nonetheless, it may result in a valid option when dealing with categorical variables, because a large amount of less frequent values, which could increase the dimensions of the resulting space, can be instead all grouped under a unique generic value (e.g., *Other*). This way, we yield just one dimension for an entire group of categorical values.

In the following Sections, instead, we will illustrate an alternative approach, where some categorical descriptors were put to good use in this complex context, without any need for dimension reduction. Rather than becoming a portion of the examples on which the learning algorithm was trained, they will be used to select the data to be presented to the learning algorithm.

## Methodology

We now present, first, some preliminary information relevant to the present study, second, a description of the methods we used to innovate our approach.

## Dataset description: type of variables, deep learning and prediction accuracy

As already mentioned, we were presented with the problem of designing a *deep learning model* able to predict the imminent failure of a device that measures water consumption in a water distribution network. Initially, we worked on a huge real-world dataset, fed with about one million mechanical water meter devices and with over fifteen million water meter *readings* of consumed water, supplied by a company that distributes water over a large area in Northern Italy. This large dataset spanned a period in time, from the beginning of 2014 to the end of 2018. To train our deep learning model, at the end of a long validation process which is described at length in [8, 9], we decided to use a smaller dataset, comprised of just those water meter devices, with at least *three valid* numerical readings. This dataset contained exactly 17,714 devices; where 15.652 were *non-defective* ones, and the remaining 2.062 were *defective*.

This dataset can be summarized by means of its eight main attributes, as reported in Table 1. Besides the first attribute, relative to the ID of the water meter, the second and third attributes are of numerical type and are used to report how much water is consumed with the passage of time (Readings and Readings Dates). The final attribute (i.e., 8) does not require any explanation.

**Table 1:** Dataset: main attributes

No	Attribute name	No	Attribute name
1	Water Meter ID	5	Meter Type ID
2	Readings	6	Manufacturer ID
3	Readings Dates	7	Type of Usage
4	Material ID	8	Labels (Faulty/Non Faulty)

Instead, attributes from 4 to 7 represent categorical information about the meter devices with the following meaning: the attribute *# 4* is the type of material of which the meter is constructed (this attribute can take on 98 different values) and from now on, we will identify this attribute with the categorical variable A. The attribute *# 5* represents the specific type of the device (this attribute can take on 45 different values). From now on, we will identify this attribute with the categorical variable B. The attribute *# 6* accounts for the manufacturer of the meter (this attribute can take on 48 different values). We will identify this attribute with the categorical variable C. The attribute *# 7* represents the type of usage of the meter (this attribute can take on 14 different values). We will identify this attribute with the categorical variable D.

Before proceeding further, we need now to verify if a correlation exists between the values that our four categorical variables (A, B, C and D) can take on and the labels we assign to the devices (i.e., *defective*, *non-defective*). In fact, if this correlation existed, there would be no need to develop a complex deep learning model to predict the failure of a given device: it would be sufficient to check if a given device either possesses or not that certain characteristic. This is, for example, the unfortunate case when all the devices in a batch, constructed of a given material (or manufactured by a certain producer) are defective.

To rule out this hypothesis, we began by developing a preliminary correlation analysis, based on the use of both the *Cramér's V* technique and the *Theil's U* index.

Starting with the *Cramér's V* technique, we tried to verify the existence of a possible statistical correlation between (the values that) each categorical variable (may take on) and the *labels* (that is, either *defective* or *non-defective*) assigned to our devices. Essentially, this method measures the association between two variables, using a *Pearson's chi-squared* statistic [27] and returning results in the continuous interval [0, 1]; where, on one side, *0* indicates no association between the investigated variables, while on the contrary *1* means that a correlation exists. It is based on the following formula: $V = \sqrt { \frac{{\chi^{2} }}{{\min \left( {k - 1, r - 1} \right)n}}} .$ In our case: $\chi^{2}$ is a *chi-squared* statistic conducted on *n*, the total number of water meter devices, *r* is the number of values that a given categorical variable may take on, and *k* is the number of different labels that can be assigned to a given device (i.e., *defective* and non-*defective*).

In Table 2, we report the results we got with the *Cramér's V* analysis we conducted for each categorical variable. As shown in Table 2, the results indicate a very low correlation between each of our categorical variables and the labels, with numerical results ranging from 0.14 to 0.32.

**Table 2:** Correlation between categorical variables and labels, using *Cramér's V index*

Categorical variables	Cramér's V
*A*/label	0.32
*B*/label	0.28
*C*/label	0.26
*D*/label	0.14

To have a further confirmation that A, B, C and D did not correlate directly with the devices of our dataset, we used also another technique, namely the *Theil's U* analysis [28]. It measures, again, the plausible degree of an association between two variables, returning a result in the continuous interval [0, 1]. *Theil's U* values can be computed based on the following formula: $U\left( {X{|}Y} \right) = \frac{{H\left( X \right) - H\left( {X|Y} \right) }}{H\left( X \right)}.$ Here, *X* and *Y* are two discrete (random) variables. In our case: *X* is one of our categorical variables, while *Y* represents the label. *H(X)* is the entropy of *X*, and *H(X|Y)* is its conditional entropy. The results of our *Theil’s U* analysis are reported in Table 3. Again, the results of Table 3 confirm a low correlation between our categorical variables and the labels, with a maximum value of just 0.13.

**Table 3:** Correlation between categorical variables and labels, using *Theil's U index*

Categorical variable	Theil’s U
*A*/label	0.13
*B*/label	0.09
*C*/label	0.09
*D*/label	0.03

What we can conclude, at this point, is that there is no statistical correlation between our categorical variables and the labels we assigned to our devices. This allows us to rule out the hypothesis that predictions can be made by simply observing the values that a categorical variable takes on, and simultaneously encourages us to continue on the road of a more complex predictive model.

At this point, we developed a deep learning model, whose main characteristics were as follows. It was based on two parallel inputs, in order to handle both the numerical time series returned by the water readings and the categorical input. The output of these two parallel branches was then concatenated and finally combined in two layers of the model to achieve the final prediction. It is worth mentioning that the complete architecture of deep learning model was already described in [8]. Rather, it is of some importance to remind that it was implemented using the *Keras* and *Tensorflow* frameworks, with *Adam* as the optimization algorithm, for eighty epochs. To measure the accuracy of the predictions, we resorted to the well-known performance metrics termed *Area Under the Curve* (AUC) of the *Receiver Operating Characteristic* (ROC).

Further, each experiment was developed in the following way. First, we used the 80% of the available devices for a traditional ten-fold cross validation procedure [29]. The results of this ten-fold cross validation procedure was given in terms of the AUC-ROC metric, and its associated standard deviation. Then, we used again the portion of 80% of the devices to train our model and, finally, we tested the prediction ability of the model with the remaining unseen 20% of data. In particular, we developed four different experiments, where respectively:

1. In the first experiment, only the available numerical values were used, that is the total amount of readings associated to our 17,714 devices;
2. In the second experiment, we used both the readings of our 17,714 devices and the categorical values mentioned above (i.e., A, B, C and D). In this case, the correspondent data were prepared using the one-hot encoding technique, with the resulting categorical space dimensionality increased up to 205. This is the result of the sum of the different types of the four categorical descriptors (98 + 45 + 48 + 14 = 205);
3. In the third experiment, we used again both the readings of our 17,714 devices and the categorical values mentioned above (A, B, C and D). Like before, the categorical data were prepared using the one-hot encoding technique, but the learning space dimensionality was decreased down to 128, by applying the *Principal Component Analysis* (*PCA*) technique [17], with a sum of variances of all individual principal components approximately equal to 90%.
4. In the fourth experiment, we used again both the readings of our 17,714 devices and the already mentioned categorical values (A, B, C and D), encoded with the one-hot encoding technique. In this case, the learning space dimensionality decreased down to 48, by virtue of the application of the *Binning* technique [26]. It is to note, here, that the 48 one-hot values used for Binning were obtained by selecting the top values for each categorical variable, according to a *Pareto* distribution (that is, appearing in the 90% of the dataset), and finally adding 1, for the “other bin” that comprised all the remaining infrequent values.

The results of these four experiments are reported in Table 4 (third and fifth columns), where the prediction accuracy of various learning models is returned in term of the AUC-ROC metric, as achieved during both the initial ten-fold cross validation phase (where also the standard deviation value is reported in the fourth column) and the testing phase.

**Table 4:** Prediction accuracy: w/o categorical variables, w/ categorical variables, PCA and Binning

Experiment	Dimension of the categorical space	AUC-ROC Ten-fold cross validation	Standard deviation	AUC-ROC testing	# of Meter Devices	
					defective	Non-defective
#1	No categorical variable	85%	1.7%	86%	2062	15,652
#2	205	78%	8.5%	83%	2062	15,652
#3	128	73%	12,5%	81%	2062	15,652
#4	48	76%	13.4%	85%	2062	15,652

If we look at the prediction accuracy value achieved without the introduction of the categorical variables (experiment #1, 86%), it is evident the impossibility to achieve a better prediction accuracy with the introduction of the additional information provided by the categorical variables (experiment #1, 83%), as a result of the increase of the learning space dimensions. Not only that, but also an attempt to reduce the dimensions of that space, using traditional techniques like *PCA* and *Binning*, either provided no real benefit (experiment #4, 85%, *Binning*) or caused a further deterioration of the prediction accuracy (experiment #3, 81%, *PCA*).

In essence, we have found a case where there is no way to obtain an improvement of the accuracy of the predictions if categorical data are added, whatever technique is employed.

At that precise point, we took the decision to adopt an alternative method which is described in the following Subsection.

## Dimension reduction with a *Pareto* analysis

Given the failure of the experiments where we tried to increase the prediction accuracy by adding categorical information, we decided to better enquire on how the values taken by each categorical variable were distributed over our 17,714 water meter devices.

**Fig. 2:**
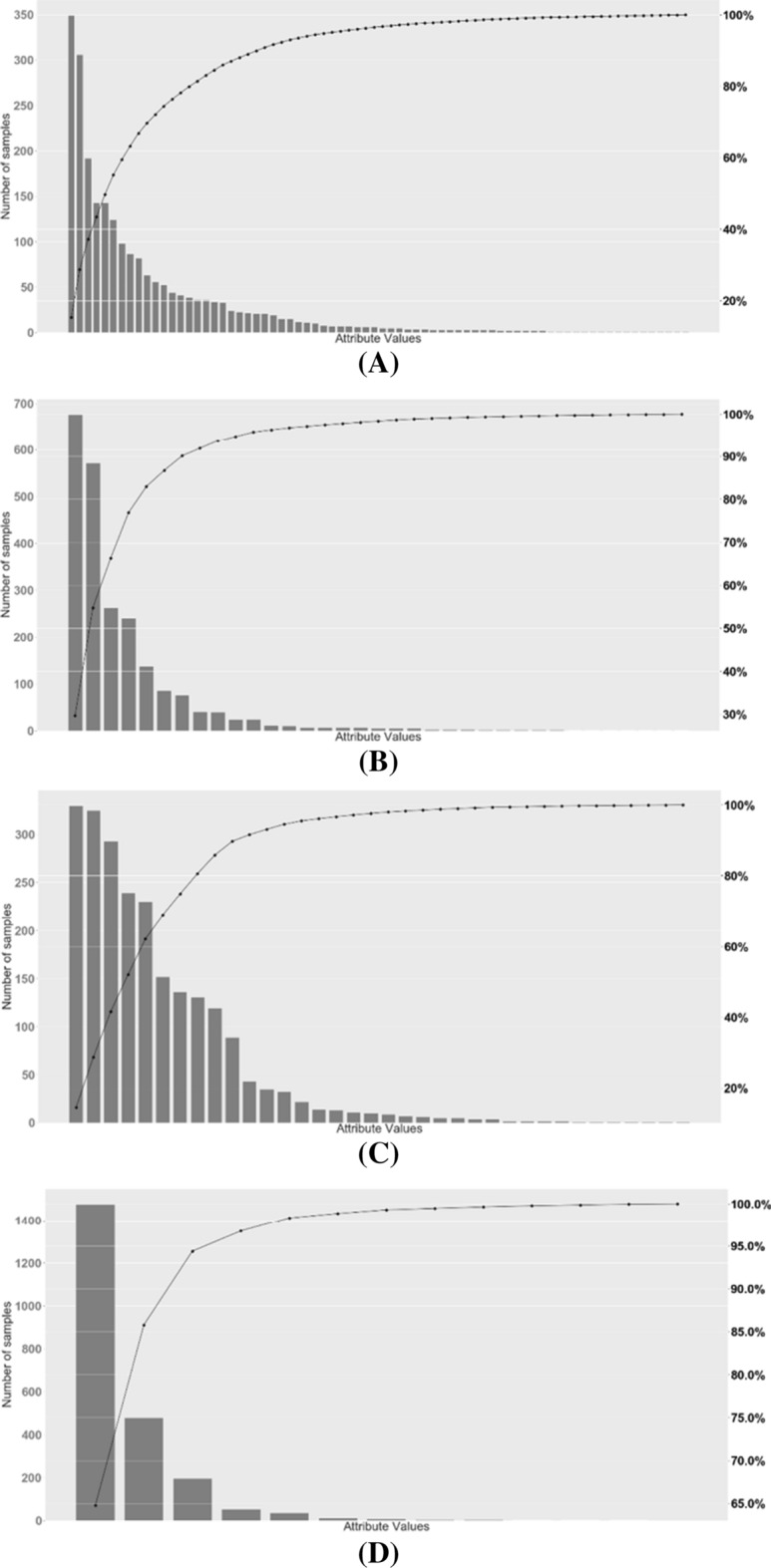
Number of devices possessing a given categorical characteristic (From top to bottom: variables A, B, C, and D)

In Fig. 2, histograms are plotted that begin to reveal an important fact. There are many devices that possess a given characteristic (or take on a specific value) of a certain categorical variable, while many other characteristics/values are scarcely relevant for those devices.

If we better analyze Fig. 2, we see that we have four plots, each one for each analysed categorical variable. From top to bottom: A, B, C and finally D. In each plot, one can read the number of devices that possess a certain characteristic (or *value*) for that categorical variable, using the scale set at the leftmost side of the *y*-axis of the Figure, while the *n* different characteristics (or *values*), for each categorical variable, are distributed over the *x*-axis. Obviously: *n* = 98 with A; *n* = 45 with B; *n* = 48 with C and *n* = 14 with D. It goes without saying that the higher is a histogram, the more are the devices possessing that given categorical characteristic.

There is another information portrayed in Fig. 2: for each categorical variable, we have a dotted curve with the cumulative percentage distribution of those *n* values over our devices. For example, if we consider the dotted curve for the *A* variable and then we look at scale set at the rightmost side of Table 2, we can recognize that the ten most frequent characteristics (out of 98) are present in almost the 90% of the devices. As an additional note: Fig. 2 has been drawn only for the defective meter devices. For the sake of conciseness, we have omitted to report an additional Figure for non-defective devices, as it would show very similar results.

At the end, a careful analysis of Fig. 2 reveals that the distribution of the categorical characteristics possessed by our 17,714 devices is shaped like a *quasi-Pareto* function [29].

As to this choice of identifying with a *Pareto* distribution the curves according to which the categorical values possessed by our devices is shaped, we could notice that there is a wide hierarchy of several other power-law or *Pareto* distributions (known, for example, as *Pareto* type I, II, III, IV and Feller–*Pareto* distributions). However, our intent, here, is simply to emphasize that our empirical observation of the curves of Fig. 2 shows that the typical 80–20 *Pareto* rule, stating that 80% of outcomes are due to 20% of causes, precisely reflect the situation under investigation. Only the adoption of a quasi-*Pareto* function fits well the trend of our four categorical variables where just a few of the most frequent values would provide a contribution in terms of knowledge representation of this phenomenon. In other words, what happens is that, given a categorical variable, just a small subset of its characteristics (or values) is possessed by the most part of the water meter devices. On the contrary, a lot of values (or characteristics) that a categorical variable can take on are not representative of any device.

Table 5 better summarizes this aspect numerically. For each categorical variable, it reports: (i) the number *n* of all the characteristics (or values) a given variable can take on, (ii) the number of the most frequently used characteristics, and finally (iii) the number of devices (both defective and non-defective) that possess those most frequent characteristics. In essence, what emerges from Table 5 is that, on average, around the 90% of meter devices possess about the 20% of characteristics (or values) of a given categorical variable.

**Table 5:** A quasi-*Pareto* distribution of the categorical characteristics

Categorical variable	# of all the characteristics	# of the most frequent characteristics	# of Meter Devices		Total number of devices remaining after the Pareto rules
			Defective	Non-defective	
A	98	23 (23%)	1855 (90%)	13,474 (86%)	15,329
B	45	7 (16%)	1854 (90%)	13,707 (88%)	15,561
C	48	11 (23%)	1889 (92%)	13,369 (85%)	15,258
D	14	3 (21%)	1945 (94%)	13,963 (89%)	15,908

By virtue of this analysis, we took the decision to reconsider how to make appropriate use of the categorical descriptors. Definitely: not as input data on which to train the model, but as relevant information to take into account to *reshape* the training dataset. In essence, the idea at the basis of our approach is that of employing the most frequent characteristics (of a given categorical variable) to select the water meter devices on which a deep learning model should be trained. Simply put, the approach can be described as follows:

1. Given a categorical variable, we train a deep learning model with just those water meter devices (and corresponding numerical readings) that possess the most frequent *characteristics* (of that variable). At the end of this first step, this strategy returns *four* deep learning models, one for each categorical variable in use. We can call them, respectively: DLM_A, DLM_B, DLM_C, and DLM_D. Obviously, each model can return a different prediction, each one with its associated accuracy.
2. At the end of point 1 above, we have four different predictions, returned by the four trained models. These predictions can be then further managed to produce a unique and final comprehensive value. Different methods can be used to this specific aim; among many alternatives, we have chosen to adopt a specific strategy, termed *bagging*, that will be better explained in a next Section (i.e., Discussion).

**Fig. 3:**
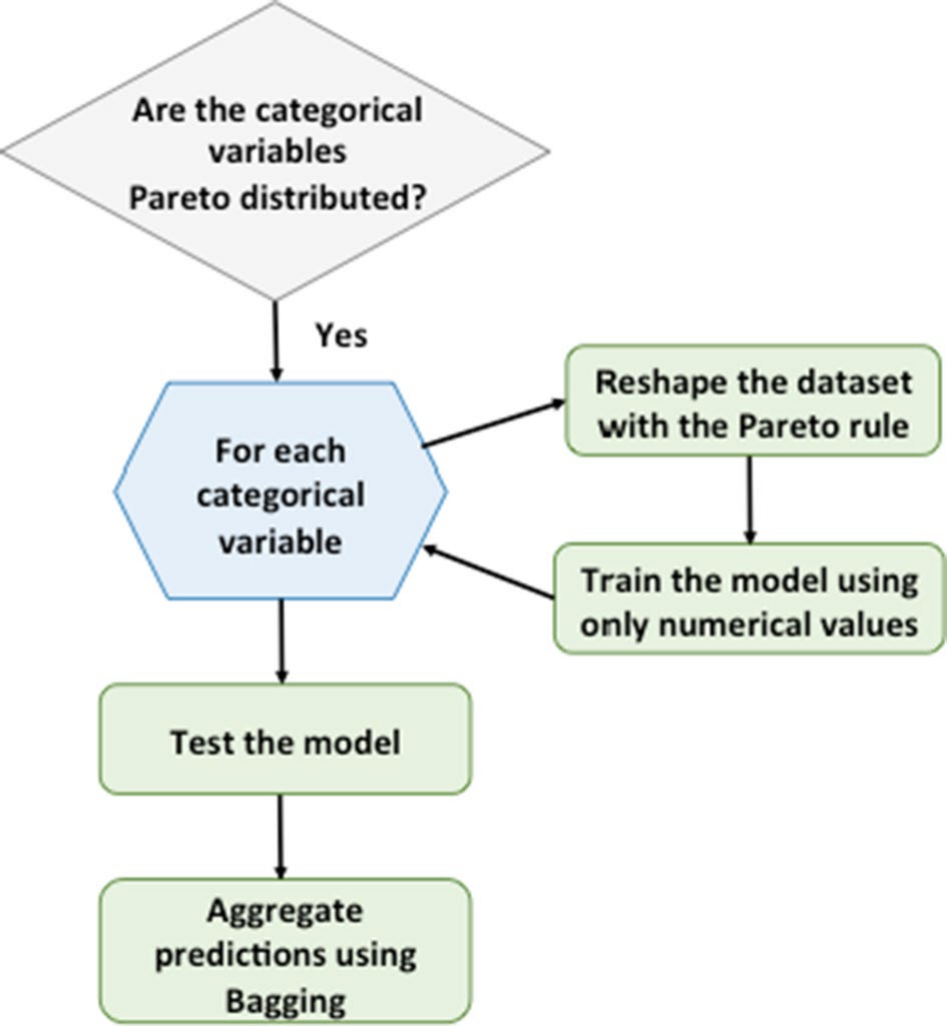
Graphical summary of the proposed methodology

To give a visual impression of our methodology, we consider of great utility the following graphical scheme, portrayed in Fig. 3, that succinctly summarizes the main steps of the *Pareto*-inspired path we have described above.

**Fig. 4:**
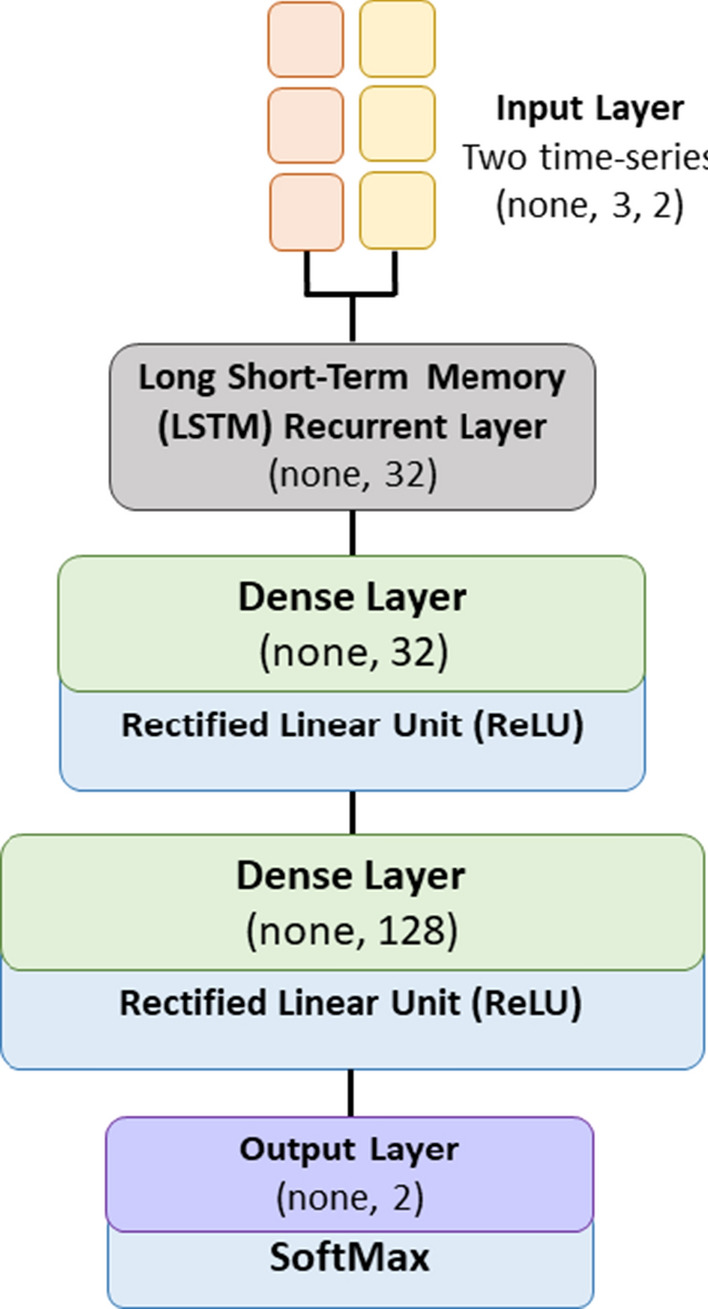
Structure of the deep learning model

Of relevant importance is also a description of the technical structure of the (four) deep learning models we have employed. We provide this description with the graphical scheme of Fig. 4, where our typical deep learning model is presented.

In particular, our typical deep learning model is comprised of the following layers: (i) an *input layer* taking as input two time-series: the former representing the water consumption measurements, and the latter representing the passage of time between consecutive readings, (ii) a Long Short-Term Memory (LSTM) recurrent layer with 32 neurons, (iii) a first Dense layer with 32 neurons, and a Rectified Linear Unit (ReLU) as its activation function, (iv) a second Dense layer with 128 neurons, and a Rectified Linear Unit (ReLU) as its activation function, (v) finally the Output layer. The Keras and the Tensorflow frameworks were used as usual to manage the above model. As per the LSTM layer, it could be interesting to note that we used the standard implementation of the LSTM layer as proposed in [30].

In addition, the following Tables 6, 7 and 8 report the hyperparameters used in our experimental setup, respectively for the Dense layers, the LSTM layer and the Adam optimizer. Not only, but it is also worth mentioning that cross-entropy was used as the loss function and each model was trained with batch size 512, for 80 epochs.

**Table 6:** Hyperparameters for the Dense layers

Hyperparameter	Value
Activation function	“ReLU”
Use bias	True
Kernel initializer	“Glorot uniform”
Bias initializer	“zeros”

**Table 7:** Hyperparameters for the LSTM layer

Hyperparameter	Value
Activation function	“tanh”
Recurrent activation	“Sigmoid”
Use bias	True
Kernel initializer	“Glorot uniform”
Recurrent initializer	“orthogonal”
Bias initializer	“zeros”

**Table 8:** Hyperparameters for the Adam optimizer

Hyperparameter	Value
Learning rate	0.001
Beta_1_	0.9
Beta_2_	0.999
Epsilon	1 × 10^−7^

To conclude this Section, there is another serious technical problem which is as follows.

Typically, when one decides to clean a given dataset, prior to subjecting it to a learning process, adequate validation procedures are to be conducted to check if the resulting dataset is coherent with the initial one.

This is exactly our case, since we need to reflect on the statistical meaning and validity of the *Pareto* operations we have carried out with the aim to reshape our initial dataset, yielding four different subsets of devices (i.e., A, B, C and D), and the correspondent learning models (DLM_A, DLM_B, DLM_C, and DLM_D).

In essence, we need to conduct an analysis to understand whether the re-adjustment of our data has transformed/altered our dataset, from a statistical viewpoint, or it has been left unaltered.

This analysis has been focused on the following facts.

Since the most important contents of our dataset consist in the water meter readings that report how much water has been consumed, per each meter device, we could conclude that no relevant statistical alteration has been made while reshaping the dataset, if the average value of the consumed water measured by all the meter devices comprised in the initial dataset is not different from the average value of the consumed water measured by the devices of all the four final subsets (A, B, C and D), obtained after the selection based on the *Pareto* rule.

In simple words, we are looking for a confirmation to the statistical hypothesis that the average quantity of consumed water, as reported in the readings comprised in the initial dataset of meter devices, is not different from that corresponding average values reported in those readings belonging to the four sets of devices, chosen after the *Pareto* selection.

Based on this idea, we conducted two different statistical tests. With the first, we assumed normal distributions (with known values for the average and standard deviation values of the consumed water) and proceeded with a *Z test*. We tested the null hypotheses (i.e., the two average values are equal) with a significance *α* factor equal to 0.01.

As seen from the results we have reported in Table 9, the null hypothesis is never rejected, i.e., we have no evidence that the average quantity of consumed water measured by the devices of the initial dataset, say µ, is different from the average quantities of consumed water measured by the devices belonging to all the four sets selected with the Pareto rule, call these averages: *µA*, µ*B*, *µC* and *µD*.

**Table 9:** Z test: Statistical coherence of the initial dataset with *A*, *B*, *C* and *D*

Test	*p-value*	α = 0.01
*µ* = *µA*	0.147059	Fail to reject the null hypothesis
*µ* = *µB*	0.037709	Fail to reject the null hypothesis
*µ* = *µC*	0.923521	Fail to reject the null hypothesis
*µ* = *µD*	0.925745	Fail to reject the null hypothesis

Not only, but we repeated the same kind of test, yet with a different statistic. Simply, we tried to use a *Student’s T test* (with an unknown standard deviation). This should be intended just as an additional attempt to confirm the previous statistical results and, in fact, not surprisingly, we got very similar outcomes, as shown in Table 10.

**Table 10:** *T* test: Statistical coherence of the initial dataset with *A*, *B*, *C* and *D*

Test	*p-value*	α = 0.01
*µ* = *µA*	0.153596	Fail to reject the null hypothesis
*µ* = *µB*	0.038466	Fail to reject the null hypothesis
*µ* = *µC*	0.926699	Fail to reject the null hypothesis
*µ* = *µD*	0.928924	Fail to reject the null hypothesis

To conclude this point, while it is true that, in general, data cleaning operations can bring to a kind of a statistical paradox, when the initial dataset is significantly different from the final one, in our case we have no statistical evidence that the most important information comprised in our dataset (the amount of consumed water) has been altered by the *Pareto* operations that have produced the four different sets of meter devices, on which our corresponding learning models have been trained.

## Results

Before showing the results got with the four deep learning models discussed before, we devote some time to provide the important following information.

The first fact to mention is that, for each model training activity, we split each subset of the meter devices (both *defective* and *non-defective*) into two separate portions. The first one consisted of the 80% of the total quantity of meter devices (devices) that were used only for the ten-fold cross validation procedure and for the training activity. Instead, the remaining 20% of devices (with their readings) were used for the testing phase. As to the metrics used for measuring the prediction results returned by our models, we made use of a set of the most popular formulas. While the most significant one remains the *AUC-ROC* metrics, whose complex definition can be read here [31], we provide a succinct definition of the other ones.

To understand them, it is fundamental the meaning of the following concepts: *TP* (or *true positives*) is the number of defective devices, predicted as defective by a prediction model; *TN* (or *true negatives*) is the number of non-defective devices, predicted as non-defective by the model; *FP* (or *false positives*) is the number of non-defective devices, erroneously predicted as defective by the model, and finally *FN* (or *false negatives*) is the number of defective devices, erroneously predicted as non-defective by the model.

Given these preliminary definitions, the following formulas (1, 2, 3, 4, 5, 6, 7) correspond, respectively, to the following concepts of *Positive Predictive Value (PPV)*, *Negative Predictive Value (NPV, True Positive Rate (TPR), True Negative Rate (TNR)*, *F1-score on Positives, F1-score on Negatives* and *Accuracy*.

1 $$ Positive\, Predictive\, Value\, \left( {PPV} \right) = \frac{TP}{{TP + FP}}, $$

2 $$ Negative\, Predictive\, Value\, \left( {NPV} \right) = \frac{TN}{{TN + FN}} , $$

3 $$ True\, Positive\, Rate\, \left( {TPR} \right) = \frac{TP}{{TP + FN}}, $$

4 $$ True \,Negative\, Rate\, \left( {TNR} \right) = \frac{TN}{{TN + FP}}, $$

5 $$ F1 - score\,on\, Positives = 2*\frac{PPV*TPR}{{PPV + TPR}}, $$

6 $$ F1 - score\, on\, Negatives = 2*\frac{NPV*TNR}{{NPV + NPR}}, $$

7 $$ Accuracy = \frac{TP + TN}{{TP + TN + FP + FN}}. $$

Another relevant fact to remind is that in our study our dataset was split over two different sets (defective and non-defective devices), whose dimensions were very different. The issue of imbalanced sets is of great relevance when they are used for training a learning model, thus needing an adequate management [32, 33]. To this aim, in all the training experiments we have carried out, we have used the traditional SMOTE technique included in the *imbalanced-learn* Python Library. This has allowed us to balance the two sets of devices, by augmenting that set of the defective ones.

The results of the oversampling operations we have carried out on the minority class (defective meters) are reported in the fourth column (SMOTE-ADDED) of the following Tables 11 and 12, for all the four learning models obtained after the application of the *Pareto* rule. Obviously, the SMOTE added devices reported in the fourth column of Table 11 are those used for the training activity, while the SMOTE added devices reported in the fourth column of Table 12 are those used for ten-fold cross validation procedure.

**Table 11:** Quantities of devices (defective and non-defective) used for training and testing plus the number of SMOTE-added devices for the minority class (defective)

Model	Train data (80%)		Smote-added	Test data (20%)	
	Non-defective	Defective		Non-defective	Defective
DLM_A	10,779	1484	9295	2695	371
DLM_B	10,966	1483	9483	2741	371
DLM_C	10,695	1511	9184	2674	378
DLM_D	11,171	1556	9615	2792	389

**Table 12:** Quantities of devices (defective and non-defective) used for the ten-fold cross validation procedure plus the number of SMOTE-added devices for the minority class (defective)

Model	Ten-fold cross validation (9 FOLDS)			Smote-added	Ten-fold cross validation (1 FOLD)	
	Non-defective	Defective			Non-defective	Defective
DLM_A	9701	1335		8366	1078	149
DLM_B	9869	1334		8535	1097	149
DLM_C	9625	1359		8266	1070	152
DLM_D	10,053	1400		8653	1118	156

Precisely, In Table 11, for each learning model, in the second and in the third columns we show the quantity of devices (non-defective and defective), used during the ten-fold cross validation procedure and the training phase, while in the fifth and in the sixth columns the number of devices is shown that were used during the testing phase. It goes without saying that each device comes with its associated readings.

Similarly, in Table 12, for each learning model, we report, in the second and in the third columns, the portion of devices (non-defective and defective), used during the training of the ten-fold cross validation procedure (i.e., 9 FOLDS), while in the fifth and in the sixth columns we report the portion of devices used during the testing of that ten-fold cross validation procedure (1 FOLD). Again, one should consider that the relative readings associated to each device.

Now, we are ready to show the results, as returned by each of the four deep learning models obtained after the application of the *Pareto* rule (namely, DLM_A, DLM_B, DLM_C, and DLM_D).

These results are shown following the path of the various procedures with which our learning model was trained. The first used one was a ten-fold cross validation procedure [34]. It is well known that cross-validation is primarily used in these cases to estimate the skill of a learning model on unseen data. That is, in order to estimate how the model is expected to perform when it will be used to make predictions on data not used during the training of the model. This is a very popular method because it is simple and because it typically results in a less optimistic estimate of the prediction accuracy than that will be returned with the train/test method. In our case, with our ten-fold cross-validation, we randomly partitioned our train data (i.e., second and third columns of Table 11) into 10 equal size subsamples. Of these 10 subsamples, 9 subsamples were used as training data (second, third and fourth columns of Table 12), while a single subsample was retained as the validation data for validating the model (columns fifth and sixth of Table 12). Then we repeated our cross-validation process 10 times (the folds), with each of the 10 subsamples used exactly once as the validation data. The 10 results from the folds were finally averaged (with also the computation of the standard deviation values). These results are reported in Table 13 using the AUC-ROC metric.

**Table 13:** Results: ten-fold cross validation

Model	AUC-ROC	Standard deviation
DLM_A	87%	1.4%
DLM_B	87%	1.4%
DLM_C	86%	1.1%
DLM_D	87%	1.5%

Encouraged by the results of our ten-fold cross validation procedure, we passed to the traditional training and testing activity, performed on the devices of Table 11. Obviously, the training phase was conducted on the 80% portion of the data (second, third and fourth columns of Table 11), while the final test was conducted on the 20% of the (unseen) data (fifth and sixth columns of Table 11). The results are provided in the terms of the metrics we have introduced above, precisely: *Positive Predictive Value (PPV)*, *Negative Predictive Value (NPV), True Positive Rate (TPR), True Negative Rate (TNR)*, *F1-score on Positives (F1 on P), F1-score on Negatives (F1 on N)*, *Accuracy* and *AUC-ROC*.

The following considerations are in order, now. First, it worth reminding that a deep learning model, typically, returns a probability value, ranging from 0 to 100%. In our specific case, the closer we are to 0%, the more is plausible the decision that that device is *non-defective*; instead, the closer we are to 100%, the more is plausible the decision that that device is *defective*. Second, we used the probability value of 0.7 as a threshold, over which a given device is definitely predicted as *defective*. The decision for the 0.7 threshold comes from the fact that this the cut-off point that allows to both maximize the true positive rate and minimize the false positive rate in the corresponding ROC curve in our specific case. Since we have employed the ten-fold cross validation procedure as the first step of our experimentation, 0.7 was computed as the average between the optimal thresholds achieved over the ten validation runs.

We would like to conclude by highlighting the fact that the prediction accuracy returned by our models, whose examples were selected with the *Pareto* rule, range from 87 to 88%. If we compare this result with the AUC-ROC value of 83% (obtained with a model trained with both the numerical and categorical variables, experiment #2, Table 4), we can observe a not negligible improvement. Nonetheless, this improvement could appear more limited if we look at other alternatives that either do not make use of categorical variables (86%, experiment #1, Table 4) or do exploit some dimension reduction procedure, like Binning, for example (85%, experiment #4, Table 4). Nonetheless, even in this case, we deem as important to have proposed a new and original method able to increase the prediction accuracy in the presence of categorical variables. Nonetheless, a more detailed discussion about the advantages and the limitations is provided developed in the Section below.

## Discussion

The present discussion aims at emphasizing both the advantages and the possible limitations of the approach we have proposed to treat categorical, high dimensional data.

First, it is important to mention that this paper starts from the consideration that many of the existing feature subset selection methods, that are commonly used for machine learning, cannot be easily extended to the case of categorical datasets, with an extremely large volume of examples.

Our proposed approach has proven to be useful in all those cases with categorical descriptors when it can be shown that the training data are distributed following a (quasi) *Pareto* statistical distribution. This should not be considered as a limitation, because the field of application may extend very far from the field we have chosen for our study (i.e., the predictive maintenance of water meter devices) up to very hot current research topics, like for example computational epidemiology [35, 36] and COVID-19 data modeling [37, 38] as well, where this kind of unbalanced statistical data distributions often occur.

Second, another intriguing issue is that it could seem that, in our training process, we have mixed notions from two different genres (feature selection using the *Pareto* rule and deep leaning). To this aim, we would like to emphasize the fact that while it is true that one of the strong advantages of a deep learning model is its inherent hierarchical feature selection along the successive level of increasing abstraction in detecting patterns, many practical situations exist where the data have huge dimensions and are also very sparse. In those cases, it becomes difficult to use a pure deep learning approach, due to the limited number of neurons typically present in the input layer. In those specific situations, a good practice can be that of using adequate projection algorithms that decrease the number of features to a reasonable number, which can be then tackled by deep learning. When this happens, we should interpret such a procedure more as a *feature extraction* procedure, rather than a feature selection, which is more typical with classical machine learning algorithms. In simple words, the *new features* that are extracted are somewhat meaningless from the point of view of the deep learning method, yet their extraction can be useful to drive the learning process, in some specific cases. This has been exactly also our case. Not only, but a new type of literature is emerging that describes similar situations, like for example in [39–41].

This latest issue has another interesting implication which can be summarized with the question if more traditional machine learning classification algorithms, like Support Vector Machines, for example, could perform better with respect to the deep learning models we have utilized selected based on the *Pareto* rule. To investigate on this subject, we have carried out an additional experiment, where more traditional machine learning algorithms were used. We employed two classical machine learning algorithms like SVM (Support Vector Machine classifier) and CART (Classification and Regression Trees), referenced respectively as [42] and [43] and developed within the context of a Python *sklearn* implementation. Results from these experiments are shown in Table 14. In particular, in the second and in the third columns of Table 14 the AUC-ROC values are reported, along with the correspondent standard deviation values, obtained with a ten-fold cross validation procedure. In the fourth column of Table 14, we show the AUC-ROC values achieved during the final testing phase.

**Table 14:** Results: Prediction accuracy with *SVM and CART*

Model	AUC-ROC Ten-fold cross validation	Standard Deviation	AUC-ROC testing
SVM w/ categorical	69%	10.4%	80%
SVM w/o Categorical	76%	2.6%	77%
CART w/ Categorical	65%	6.3%	73%
CART w/o Categorical	74%	2.7%	74%

As it is evident from a comparison of these results with those from Table 15, traditional machine learning algorithms have provided, in our case, prediction accuracy performances that are worse than that obtained with the method proposed in this paper, thus confirming the validity of our choices.

**Table 15:** Results: testing

Model	PPV	NPV	TPR	TNR	F1 on P	F1 on N	Accuracy	AUC-ROC
DLM_A	36%	96%	74%	83%	49%	89%	82%	88%
DLM_B	43%	96%	70%	88%	54%	92%	86%	87%
DLM_C	39%	96%	73%	84%	51%	90%	83%	88%
DLM_D	45%	96%	70%	88%	55%	92%	86%	87%

Third, it is important to provide an answer to a more practical question that could emerge at this point of the discussion: How can our method be serviceably if applied to any of the meter devices that are utilized to measure water consumption in our case? In other words, if we have to make a prediction on a *new meter device*, how can we proceed? The answer to this question relies on the simple application of the following procedure. We should, first, consider that device, and check if it possesses the categorical characteristics of either the variable A or B or C or D. If that device possesses any of the categorical characteristics of interest, we can use the corresponding model (either DLM_A, or DLM_B, or DLM_C, or DLM_D) to make our predictions. Instead, in the negative case, we should not use our models to make a reliable prediction for that device, and we should resort to a more traditional approach.

However, it should be noticed that the likelihood that a device does not possess any of those characteristics, at least in the context of the dataset we have studied, is quite low; i.e., below 10% on average, as our *Pareto* analysis has demonstrated.

Fourth and final. The approach we have proposed can be seen as a method that can be used in combination with additional techniques, useful to improve its predictive performances. Think of the *Bagging* technique, for example [44]. We could use it, in fact, whenever we have a device that possesses the characteristics of all the four models together, in the following way. We could use each different model, in isolation, to return its prediction results for that given device, and then we could average over all those returned results, to produce a unique and comprehensive prediction. Obviously, we can expect that this *Bagging* strategy, at least with our dataset, can work only for a limited quantity of meter devices; yet it could provide finer predictions, whenever applicable.

Following this reasoning and to conclude this Section, we have tried to test the aforementioned *Bagging* strategy on that subset of our meter devices (both *defective* and *non-defective*) that possessed the categorical characteristics of all the four models (DLM_A, DLM_B, DLM_C, and DLM_D). This intersection counted 2304 non-defective devices and 313 defective ones. The results of this final testing experiment are shown in Table 16. As shown in Table 16, we have an improvement of the most important metrics, especially Accuracy and AUC-ROC that has increased up to the value of **90%**.

**Table 16:** Testing results with Bagging

Model	PPV	NPV	TPR	TNR	F1 on P	F1 on N	Accuracy	AUC-ROC
DLM/Bagging	65%	96%	61%	83%	63%	95%	91%	90%

## Conclusions

We have developed a *deep learning model* able to predict if a device that measures water consumption in a water distribution network is either defective or not. The model was based on both the measurements of consumed water and on the categorical (technical) characteristics that a device possesses.

The novelty of our approach rests upon the idea of exploiting those categorical characteristics to better select the sets of devices on which the model goes trained.

Avoiding the use of those categorical characteristics as a direct input to the model has removed the danger of an explosion of the dimensions of the learning space, and with this approach we have reached predictive accuracies ranging from 87 to 90%, for an amount of 90% of the available devices.

The approach we have proposed was devised to select the training data based on a *Pareto* analysis conducted on the categorical descriptors, thus avoiding the explosion of the data space dimensions while keeping intact the statistical coherence of the portion of the dataset selected for training.

We have provided empirical evidence that this approach maintains its validity even if compared with more traditional space dimension reduction methodologies and classical machine learning algorithms.

## Data Availability

The datasets used and/or analyzed during the current study are available from the corresponding author on reasonable request.

## Abbreviations

DL: Deep learning
DLM: Deep Learning Model
PCA: Principal correspondence analysis
CA: Correspondence analysis
*CATPCA*: Categorical principal components analysis
MDS: Multi-Dimensional scaling
AUC ROC: Area under the curve receiver operating characteristic
LSTM: Long short-term memory
ReLU: Rectified linear unit
TP: True positives
TN: True negatives
FP: False positives
FN: False negatives
PPV: Positive predictive value
NPV: Negative predictive value
TPR: True positive rate
TNR: True negative rate
F1 on P: F1-score on positives
F1 on N: F1-score on negatives
SVM: Support vector machines
CART: Classification and regression trees
